# Investigation of Patient-Centric 3D-Printed Orodispersible Films Containing Amorphous Aripiprazole

**DOI:** 10.3390/ph15070895

**Published:** 2022-07-19

**Authors:** Ju-Hyun Lee, Chulhun Park, In-OK Song, Beom-Jin Lee, Chin-Yang Kang, Jun-Bom Park

**Affiliations:** 1 College of Pharmacy, Sahmyook University, Seoul 01795, Korea; hyun102@boryung.co.kr (J.-H.L.); iosong74@hanmail.net (I.-O.S.); kangjy@syu.ac.kr (C.-Y.K.); 2 College of Pharmacy, Jeju National University, Jeju 63243, Korea; chulhunp1020@gmail.com; 3 College of Pharmacy, Ajou University, Suwon 16499, Korea; bjl@ajou.ac.kr; 4 Bioavailability Control Lab, Sahmyook University, Seoul 01795, Korea

**Keywords:** hot-melt extrusion, 3D printing, aripiprazole, puncture strength, orodispersible film, patient-centric drug delivery

## Abstract

The objective of this study was to design and evaluate an orodispersible film (ODF) composed of aripiprazole (ARP), prepared using a conventional solvent casting technique, and to fuse a three-dimensional (3D) printing technique with a hot-melt extrusion (HME) filament. Klucel^®^ LF (hydroxypropyl cellulose, HPC) and PE-05JPS^®^ (polyvinyl alcohol, PVA) were used as backbone polymers for 3D printing and solvent casting. HPC-, PVA-, and ARP-loaded filaments were applied for 3D printing using HME. The physicochemical and mechanical properties of the 3D printing filaments and films were optimized based on the composition of the polymers and the processing parameters. The crystalline states of drug and drug-loaded formulations were investigated using differential scanning calorimetry (DSC) and powder X-ray diffraction (XRD). The dissolution and disintegration of the 3D-printed films were faster than those of solvent-cast films. HPC-3D printed film was fully disintegrated within 45 ± 3.5 s. The dissolution rate of HPC films reached 80% within 30 min at pH 1.2 and pH 4.0 USP buffer. There was a difference in the dissolution rate of about 5 to 10% compared to PVA films at the same sampling time. The root mean square of the roughness (Rq) values of each sample were evaluated using atomic force microscopy. The higher the Rq value, the rougher the surface, and the larger the surface area, the more salivary fluid penetrated the film, resulting in faster drug release and disintegration. Specifically, The HPC 3D-printed film showed the highest Rq value (102.868 nm) and average surface roughness (85.007 nm). The puncture strength of 3D-printed films had desirable strength with HPC (0.65 ± 0.27 N/mm^2^) and PVA (0.93 ± 0.15 N/mm^2^) to prevent deformation compared to those of marketed film products (over 0.34 N/mm^2^). In conclusion, combining polymer selection and 3D printing technology could innovatively design ODFs composed of ARP to solve the unmet medical needs of psychiatric patients.

## 1. Introduction

Three-dimensional (3D) printing technology is a versatile technique in the pharmaceutical field because it can produce various pharmaceutical formulations and customize medicines for patients [1,2]. These advantages can be a driving force for developing new treatments and improving the safety and efficacy of pharmaceuticals [3]. In August 2015, SPRITAM^®^ (levetiracetam), the first drug manufactured by 3D printing, was approved by the United States Food and Drug Administration (FDA). A fast-disintegrating formulation with a porous structure was fabricated using the ZIPDOSE technology. After FDA approval was granted for this drug, 3D-printed medicines gained interest in the pharmaceutical field [4,5]. The 3D printing process can be categorized into binder jetting and material extrusion. Although the printing process differs depending on the printing style, there are basic steps for designing the shape and structure of the final drug product using software such as computer-aided design (CAD). CAD converts a file into a printer-readable format by processing the raw material according to the desired printing style. After printing is completed, post-processing steps such as drying and polishing are performed using selective laser sintering or polymerization [3].

The manufacturing process, known as fusion deposition modeling (FDM) or fused filament fabrication (FFF), selectively extrudes a material through a nozzle or hole mounted on the equipment head. The FDM printing produces layer-by-layer units and prints using filaments [3,6]. However, commercial filaments are not loaded with drugs and are, therefore, not applicable in generating pharmaceutical products. Hot-melt extrusion (HME) can be used to manufacture drug-loaded filaments [7]. Especially, the low-dosed drug-loaded filament could achieve good content uniformity and precise microdosing control for improving patient compliance [8,9]. It can be applied to various technologies other than filament manufacturing, such as solubilizing poorly soluble drugs, taste masking bitter drugs, and controlled release drug delivery platforms [10,11,12]. Not all polymers can be used for 3D printing because the filaments used in FDM should not be easily broken or bent by physical manipulation [7]. In fact, 3D printed formulations can effectively control the desirable strength and hardness to prevent the abuse of physical manipulation and technical issues in the manufacturing process [13,14]. Suitable 3D-printing polymers include cellulose derivatives such as hydroxypropyl cellulose (HPC), hydroxypropyl methylcellulose (HPMC), and polymethacrylates (Eudragit L, R.L., and RL-PO), polyvinyl alcohol (PVA), polyethylene glycol acetate, and Soluplus^®^ [11,15,16]. These polymers can be processed into thin films, implants, and tablets using FDM methods [17,18].

Orodispersible films (ODFs) are thin drug-loaded films prepared for rapid disintegration of a drug in the oral cavity when placed on the tongue [19]. It is an easy-to-administer oral medication for pediatric and geriatric patients with difficulty swallowing oral medications [20]. Since this formulation offers a high degree of convenience for taking medications, many recent studies have focused on patient-focused drug development, including optimization of ODF formulations, taste masking, and the mechanical properties and characteristics of drug delivery systems [21]. A general method for manufacturing ODFs is solvent casting, manufacturing a film by dissolving a drug and polymer in an organic solvent, mixing, and drying [22]. The 3D printing method can produce films without using organic solvents. It is a promising alternative ODF manufacturing method as it eliminates the toxicity and environmental problems associated with organic solvents [23,24].

In this study, different ODFs were manufactured by 3D printing and solvent casting methods using the Biopharmaceutics Classification System (BCS) class II drug aripiprazole (ARP) that treats schizophrenia. In terms of drug and microenvironmental modifications, there was some research on changing salt forms and pH-dependent self-emulsifying formulations of the ARP [25,26,27]. Salts forms and lipid-based surfactants are not free from fluctuating bioavailability issues due to pH variability in the human body pH [28]. A few studies have been performed on 3D printed dosage forms of ARP [29,30]. The limitations of these studies are that the developed 3D-printed formulations used only a single polymer. Additionally, the dissolution mechanism of the optimized formulation has not been elucidated by considering the critical quality attributes involved in continuous manufacturing. Our study can provide future development by offering a holistic approach to the dissolution mechanism based on the mechanical properties of 3D-printed solid dosage forms. The mechanical strength and surface morphology of the 3D-printed formulation may synergize the water penetration with the dissolution mechanism.

The films were prepared using different film-forming polymers, including Klucel^®^ LF (HPC) and PE-05JPS^®^ (PVA) [31]. Additionally, two film-forming polymers have been used for application in FDM. The 3D-printed filaments were manufactured using HME technology. The physicochemical properties of ARP in drug-loaded filaments and films were determined using differential scanning calorimetry (DSC) and powder X-ray diffraction (PXRD). The film formulation characteristics of the ODFs were evaluated by optical microscopy and atomic force microscopy (AFM). AFM revealed the surface roughness parameters of the microscopic topography of the ODFs. The puncture strength of the ARP-loaded ODFs was determined using a texture analyzer. The mechanical stability of the ARP-loaded ODFs, relevant to handling post-manufacturing film formation, was investigated by comparing the puncture strength. The ARP dissolution from the films was evaluated in USP buffer media (pH 1.2, 4.0, and 6.8) using the paddle method following the U.S. Pharmacopeia [32,33].

## 2. Results and Discussion

### 2.1. HME Filament and 3D Printing

Drug-loaded filaments can be thermodynamically molten or denatured in a customizable process to form 3D structures. Table 1 summarizes the composition and processing parameters of each drug-loaded filament. The ARP content of filaments prepared by the HME process was 10% *w*/*w* compared with those prepared by the impregnation technique. Significantly, the primary polymer with HPC and PVA was set to 85 (*w*/*w*%) in formulation composition. The carrier polymers were primarily used in the range of 50% up to 95% [34]. The rest of the excipients would be the drug, plasticizer, salivary stimulating agent, and processing promoters. Citric acid would effectively act as a solid-state plasticizer in the range of 2.5% up to 10 (*w*/*w*%) [35]. A low concentration of sucralose is preferred as a sweetener in HME products. Other natural glucose and fructose were decomposed at hot-melting processing temperature [36].

**Figure 1:**
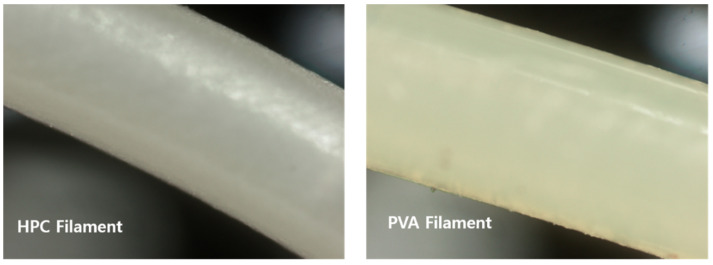
Photographs of the hydroxypropyl cellulose filament and polyvinyl alcohol filament.

As shown in Figure 1, the HPC filaments appeared opaque and white. The PVA filaments were opaque and yellow. Both HPC and PVA filament thicknesses were 1.75 ± 0.05 mm. One layer of film thickness was 0.1 mm. During the 3D printing process, if the build plate level was not horizontal, the film was not densely formed during the manufacturing process. The preparation time of the 3D-printed film was 65 s. Specifically, the film-forming HPC and PVA produced filaments with a constant thickness and smooth surface via HME. It was assumed that there was no drug recrystallization from the filament surface.

HME with 3D printing technologies could provide an innovative approach for developing immediate-release dosage forms [37]. Owing to the fusion system of HME and 3D printing technology, the preparation of drug-loaded filaments can be multifaceted to improve the solubility and stability of the active pharmaceutical ingredient (API). When 3D printing is used, the filament should have suitable hardness and flexibility, and the drug content of the final product should be uniform when the filament thickness is constant [38]. HME technology is a versatile tool for customizing drug-loaded filaments. HME has been used to prepare amorphous solid dispersions to increase the dissolution rate of APIs with poor water solubility [39].

The thermodynamic process of the HME can ultimately convert the physical state of the ARP. Additionally, highly hydrophilic polymers, forming suitable filaments with high puncture strength and modulus, can be applied in the 3D-printed solid-dosage form manufacturing process. Therefore, using HME filaments can reduce the processing steps required to manufacture pharmaceutical dosage forms. Hydrophilic polymers’ crystallinity and mechanical properties can affect dosage-form surface roughness and drug release profiles. These properties can design immediate-release solid dosage forms [40].

### 2.2. Physicochemical Properties of the ODFs

**Figure 2:**
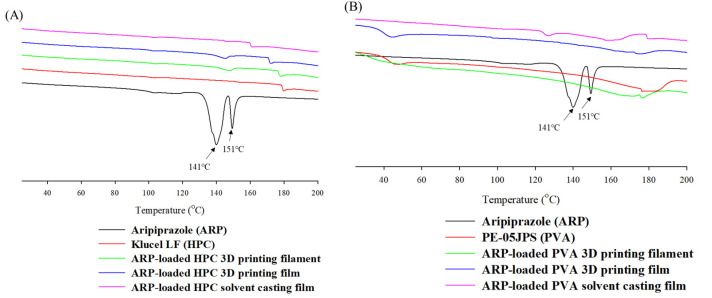
DSC thermograms of the aripiprazole and developed formulations in (**A**) hydroxypropyl cellulose polymer and (**B**) polyvinyl alcohol polymer.

As shown in Figure 2, endothermic peaks of pure ARP were observed at 141 and 151 °C, corresponding to the phase transition and melting points of crystalline states [41]. Two crystalline peaks were correlated with crystalline form III and form I [42]. No endothermic peak was observed in the DSC thermograms of the PVA-based filaments and films manufactured through the HME process. Specifically, no endothermic melting peak of ARP in the PVA-based formulations was observed in the films manufactured by the solvent casting method. The HPC-based ARP-loaded films prepared by 3D printing exhibited partial endothermic peaks at approximately 150 °C. The recrystallization of the ARP could have partially happened in 3D-printed filaments and films. The DSC thermograms of the ARP solvent casting ODFs displayed no endothermic peak within the processing temperature range. These results indicated that amorphization of the developed ARP films progressed in the manufacturing process.

**Figure 3:**
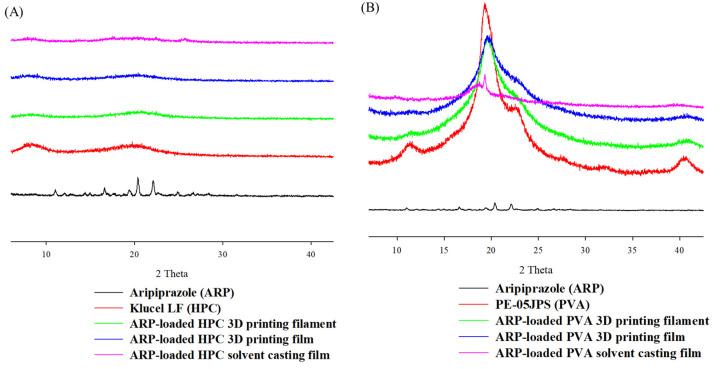
X-ray diffraction patterns of the aripiprazole formulations in (**A**) hydroxypropyl cellulose polymer and (**B**) polyvinyl alcohol polymer.

As shown in Figure 3, XRD patterns showed sharp crystalline peaks between 10° and 30° for crystalline ARP. Specifically, the XRD pattern of ARP exhibited sharp crystalline peaks at 10°, 16°, 18°, 20°, 22°, and 24°. The characteristic peaks of ARP did not appear in the 3D-printed or solvent-cast films. Additionally, the XRD patterns of the ARP-loaded PVA and HPC filaments manufactured by HME exhibited no peaks, indicating the amorphous state of ARP in both filaments. The amorphization of ARP was successfully finished regardless of the preparation method of films. Depending on the resolution of the measurements, the crystalline phase of ARP is mostly shifted by the manufacturing process. A physical state change of ARP is feasible because the significant peak entirely disappeared in developed film formulations.

Based on the DSC and XRD analysis results, all films and filaments demonstrated a change in ARP from a crystalline structure to an amorphous state [43]. ARP changed entirely to an amorphous state and was dispersed uniformly in the carrier. The amorphous filaments were melted using FDM to produce a film. The FDM technique uses an air compression barrel and nozzle in a single step. The amorphous form of ARP was maintained in the HPC and PVA films.

### 2.3. Mechanical Properties of the ODFs

A texture analyzer was used to evaluate the puncture strength of all film samples. The upper part of the film with a flat and smooth surface was used to minimize the influence of the particle surface during measurement. As summarized in Table 2, the thickness of ARP-loaded films was around 0.11 to 0.12 mm. Their thickness was comparable to that of the SmartStrip (0.15 ± 0.02 mm). The mechanical properties of ODFs can play a critical role in the physical uniformity and stability of the dosage forms [44]. The puncture strength of 3D films is a critical parameter for product quality. The mechanical strength and material porosity were significantly affected by the processing parameters. The films should be robust and flexible to avoid rupture during processing, packaging, transportation, and handling [19].

PVA films exhibited a higher puncture strength, indicating that the PVA films were more flexible and durable than the HPC films. Specifically, the mean values of the puncture strength of the HPC-based films were lower than those of the PVA-based films. The puncture strength of all films was about 8 to 14 times higher than the puncture strength of 0.08 N/mm^2^ required by the processing criteria of pharmaceutical film. The puncture strength of PVA-SC films exhibited the highest value of 1.12 ± 0.42 N/mm², indicating that its durability was more robust than any other film. Additionally, the PVA 3D film has better puncture strength than the HPC 3D film. PVA vinyl polymer could have strong mechanical strength in the same manufacturing process due to the atactic material maintaining crystallinity compared to the cellulose-based HPC semi-crystalline polymer with amorphous domains.

**Figure 4:**
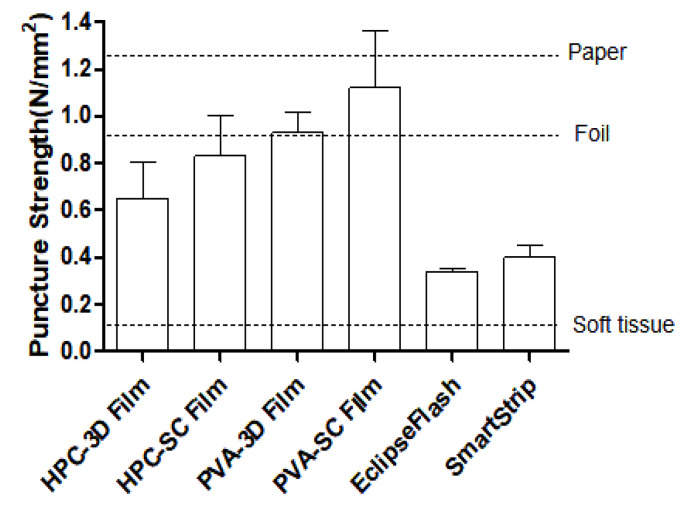
Puncture strength of the marketed products and film samples (dashed lines represent the reference materials).

Interestingly, the puncture strength of the solvent-cast films was relatively higher than that of 3D-printed films based on the same polymer. In completing the process fill density of the solvent-cast film formulations, the structural relaxation of the solvent-casting solution occurred uniformly through the solid-state plasticizer. Therefore, these results suggested that manufacturing processes significantly affected the mechanical strength of the film. It has been proven that the puncture strength of the film must be at least 0.08 N/mm² to manage the film without inducing damage in the manufacturing process [45]. Through the chart bar graph in Figure 4, it was confirmed that all the films had the strength to prevent deformation of the film shape compared to the puncture strength of commercially available films, soft tissue, foil, and paper.

### 2.4. Morphological Examination of the ODFs

**Figure 5:**
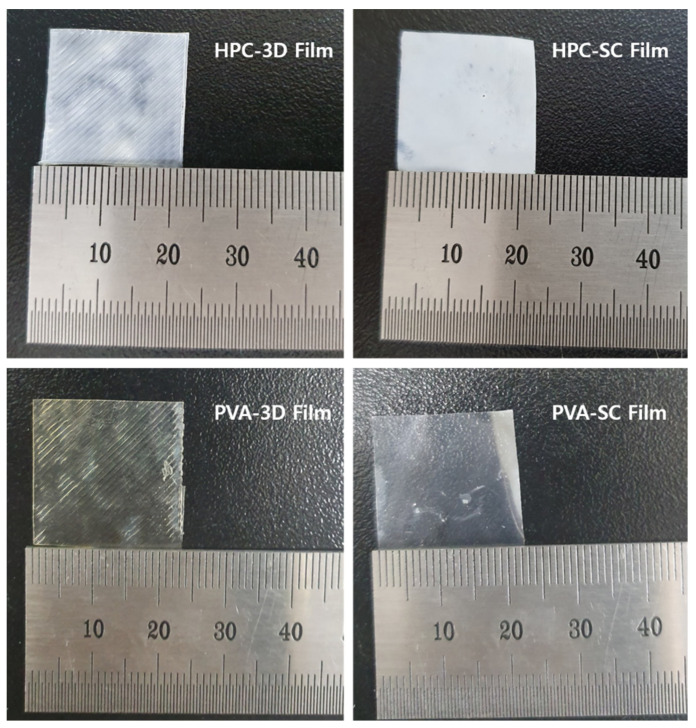
Photographs of the 3D-printed films and solvent-cast films.

When the surface of the film was visually observed, both the 3D and SC films, manufactured using HPC and PVA as polymers, appeared to be homogeneous (Figure 5). Specifically, the film surface was observed under an optical microscope at a magnification of 40×, and the 3D-printed films were printed using a layer-by-layer technique.

**Figure 6:**
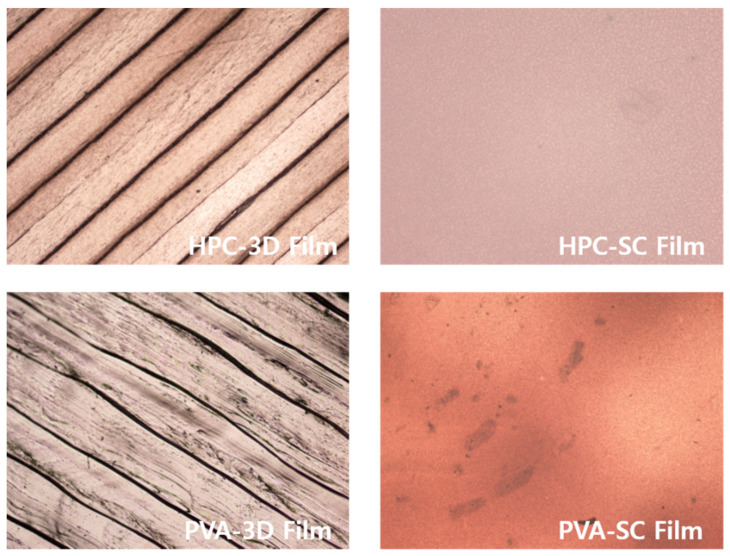
Optical microscopy images of the 3D-printed and solvent-cast films.

**Figure 7:**
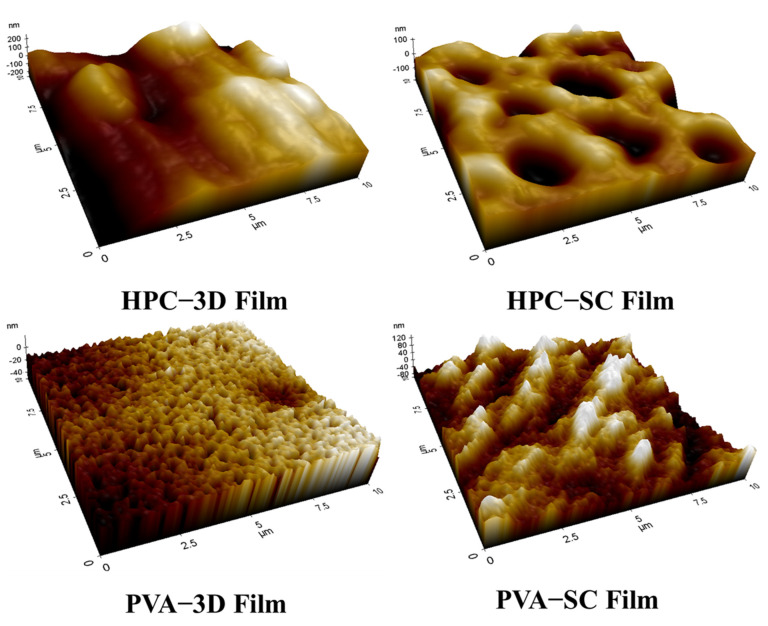
Atomic force microscopy topography of the 3D-printed (3D) films and solvent-cast (SC) films.

The interlayer spaces and the porosity of 3D-printed films were generated by the layering process of 3D printing (Figure 6). Scanning by AFM determines the roughness of a sample surface, visualizing the roughness of the sample surface that can be expressed as the root mean square (RMS) of the roughness (Rq) value of the 3D-printed and solvent-cast films (Figure 7).

The surface roughness values of the ODFs are listed in Table 3. The HPC 3D-printed film exhibited the highest Rq value (102.868 nm). The average surface roughness of the PVA 3D-printed film was 27.748 nm. According to the manufacturing method, the Rq of the 3D-printed films (HPC: 102.868 nm, PVA: 35.025 nm) was higher than that of the solvent-cast films (HPC, 44.310 nm; PVA, 12.117 nm). For the polymer type, the Rq value of the HPC films was higher than that of the PVA films.

The filling density and robustness of the films differed depending on the manufacturing method. When the films were manufactured using the solvent casting method, no porosity was observed, owing to the continuous processing of the film structure. The filling density of the solvent-cast film layers was higher than that of the 3D-printed layers. These results were observed during the surface morphological examination of the 3D-printed films (Figure 5, Figure 6 and Figure 7).

Figure 6 and Table 3 indicate that the ARP-loaded HPC 3D-printed film exhibited more porosity and rougher surface than the other types. The higher the Rq value, the rougher the surface, and the larger the surface area, the more saliva can penetrate the film, facilitating faster drug release and disintegration [46]. The 3D-printed films had a larger surface area than solvent-cast films. The dissolution medium can easily penetrate the layers of the film, and the drug can quickly dissolve [47,48,49]. The pore formation and surface roughness of ARP-loaded ODFs affected their drug release profiles.

As a result, the porosity of polymer-based drug-loaded films significantly facilitated the rapid disintegration and dissolution process with higher surface roughness [50]. Although traces of water drops appeared on the solvent-cast film, the surface did not show any pores owing to the continuous film-manufacturing process. The 3D-printed films showed higher porosities and larger surface areas than the solvent-cast films. The surface areas of the 3D-printed films were broader than those of solvent-cast films. Additionally, the differences in film surface roughness between the different methods and compositions of the films varied their disintegration and dissolution. Therefore, the 3D-printed films prepared using HPC exhibited a faster disintegration and drug release.

### 2.5. Disintegration Time and Dissolution Profiles of the ODFs

The films were disintegrated using the Petri dish method [51]. According to USP, fast-dissolving tablets should disintegrate within 60 s in a bioequivalence medium. However, there are no official guidelines for the disintegration time of ODFs [52]. HPC films have a rougher surface than PVA films. The bioequivalent medium could easily penetrate the film structure. As summarized in Table 2, the disintegration of the 3D-printed films was faster than that of solvent-cast films made from the same polymer. Specifically, the HPC-3D printed film disintegrated within 50 s (45 ± 3.5 s). The filling density determined the structural relaxation and mechanical strength of the film according to the manufacturing process and types of the polymer [53]. The water permeation of the biological fluids further accelerated the structural relaxation of film, resulting in rapid disintegration. The shorter disintegration time of the ODFs indicated that the dissolution rate of the drug would increase when placed in the oral cavity. Thus, masking the bitter taste of ODFs may effectively improve patient medication adherence.

**Figure 8:**
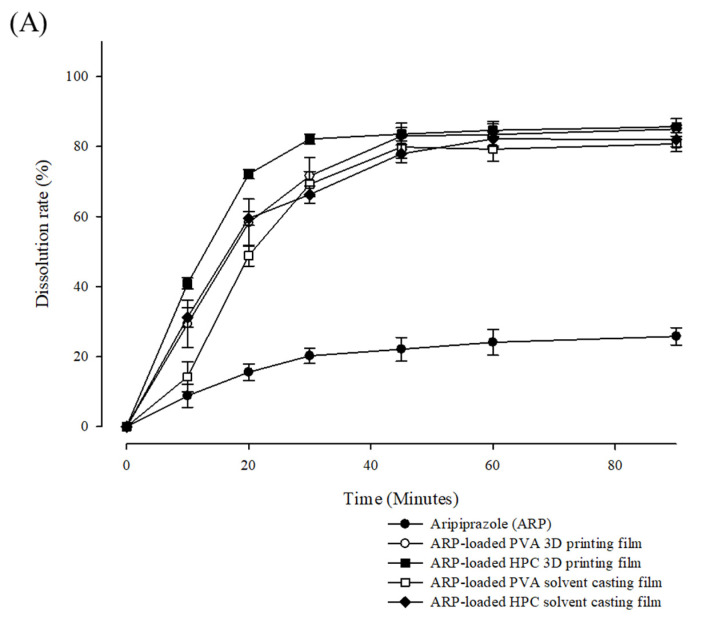

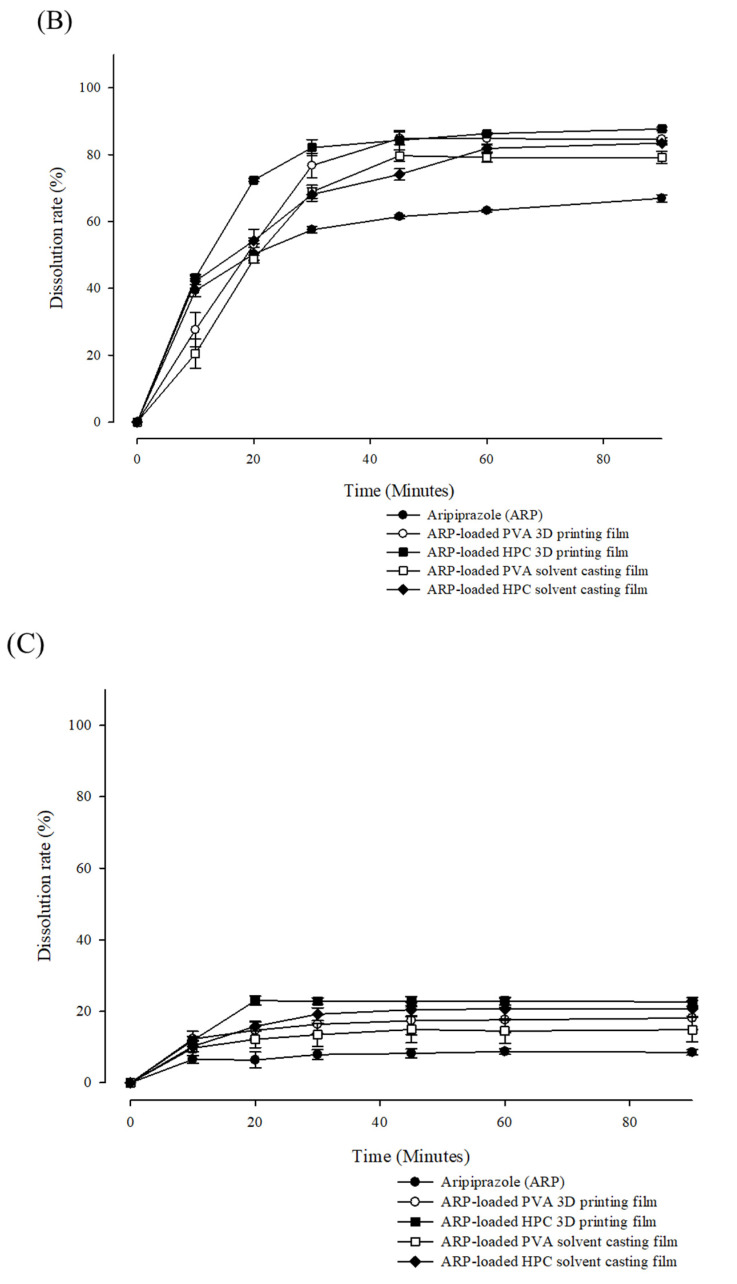
Dissolution profiles of aripiprazole (ARP) and ARP-loaded film formulations in different bioequivalent mediums, (**A**) pH 1.2 USP buffer, (**B**) pH 4.0 USP buffer, and (**C**) pH 6.8 USP buffer (*n* = 3).

The dissolution rate of the ARP films was evaluated in pH 1.2, pH 4.0, and pH 6.8 USP buffer mediums (Figure 8). As ARP changed from a crystalline structure to an amorphous structure through the HME manufacturing process, the solubilities of ARP in the 3D-printed and solvent-cast films were higher in the pH 1.2 and pH 4.0 buffers than that of the crystalline ARP. For the 3D-printed and solvent-cast films using HPC and PVA, the difference in the maximum dissolution rate was less than 5% in all buffer media. There was no significant difference in the maximum dissolution rate of the ARP in films made by different manufacturing methods. However, there was a significant difference in the release rate in the initial stage depending on the manufacturing method used. The release rate of ARP from the 3D-printed films was faster than that from the solvent-cast films at the initial stage.

Because of faster erosion, the films prepared using HPC dissolved faster than those prepared using PVA. Furthermore, the surface area was larger in the HPC films than in the PVA films, owing to the high roughness of the HPC films [54]. ARP and all films exhibited a low dissolution rate at pH 6.8, suggesting that ARP has pH-dependent solubility. Maintaining the pH-dependent solubility of API with citric acid can provide a taste-masking effect without releasing bitter drugs in the oral mucosa [55]. Notably, the dissolution rate of ARP-loaded HPC 3D printed films rapidly reached the highest point within 30 min (pH 1.2; 82.15 ± 1.36%, pH 4.0; 82.11 ± 2.38%, and pH 6.8; 22.85 ± 0.89%). Consequently, the 3D-printed film using HPC achieved a rapid disintegration and an initial dissolution rate.

## 3. Materials and Methods

### 3.1. Materials

ARP was obtained from TEVA Tech (Parsippany-Troy Hills, NJ, USA). HPC (Klucel^®^ LF) was provided by Ashland Global Specialty Chemicals Inc. (Anaheim, CA, USA), and PVA (PE-05JPS^®^) was provided by JVP (Osaka, Japan). Sucralose was used to mask the ARP’s unpleasant taste. Citrate was used as a saliva-stimulating agent. All the chemicals and solvents used were of analytical grade.

### 3.2. Preparation of Filaments and Film Using the 3D Printing Method

ARP, film-forming polymer, sucralose, and citrate were mixed to prepare the drug-loaded filaments using a Pharma 11 twin-screw extruder system (Thermo Fisher Scientific, Waltham, MA, USA). The processing parameters and compositions of the HMEs are listed in Table 1. The diameter of the die in the extruder was set as 1.5 mm.

The ODFs were prepared using a Y1010 3D printer (PMAX, Haikou, HI, China). The shape and size of the ODF were designed through the Rhinoceros^®^ software version 6 (Robert McNeel & Associates, Seattle, WA, USA). The printing path width and height were performed within the product forming size range 110 × 110 × 110 mm (W × D × H). The dimensions of the film were 22 × 22 × 0.1 mm (W × D × H), and the design file was exported in the stereolithography (STL) file (.STL) format. The STL file was then converted into a G-code file using the Cura^®^ slicer program. The printing nozzle diameter of this 3D printer was set to 0.4 mm. Engraving accuracy could be 0.1 mm. XY-axis positioning accuracy could be 0.011 mm. Additionally, Z-axis positioning accuracy would be 0.0025 mm. The outer wall thickness was set to 0.15 mm, and the density of the inner filling was 100%. The processing temperatures of the HPC 3D-printed and PVA 3D-printed films were 130 and 200 °C, respectively. The printing speed was 60 mm/s.

### 3.3. Preparation of Film Using the Solvent Casting Method

The composition of the solvent casting film was the same as that of the HME 3D printing film formulation. ARP and film-forming polymers, including HPC and PVA, were dissolved in dimethyl sulfoxide (DMSO) using a stirrer at 100 rpm for 30 min. The solutions were then gently poured into a thin Petri dish and dried in an oven at 65 °C for 12 h. After drying, the film was cut into 22 mm × 22 mm pieces.

### 3.4. Physicochemical Properties of the ODFs

DSC and XRD analyses were performed to evaluate the physicochemical properties of the ARP in the 3D-printed and solvent-cast films. The thermodynamic profiles of raw ARP and developed films were analyzed using a Q2000 differential scanning calorimeter (TA Instruments, New Castle, DE, USA). The temperature of the sample was increased with an argon atmosphere (50 cm^3^/min) from 25 to 200 °C at a rate of 10 °C/min. Measurements were performed in a hermetically sealed aluminum pan. Melting points and phase transition points were determined as the onset of the peaks.

The XRD patterns of raw ARP, polymers, and developed films were analyzed using a D8-ADVANCE X-ray diffractor (Bruker, Billerica, MA, USA) equipped with a PSD detector VÅNTEC and CuKα1 radiation. The instrument was performed in the 2 theta range at 5–42.5° with the scan step 0.02° and scan speed 0.5°/min.

### 3.5. Morphological Examination of the ODFs

Optical microscopy and AFM were used to identify the morphological characteristics of the 3D-printed and solvent-cast films. The thickness of the films was measured using a Vernier caliper. The scan size of the atomic force microscope (ParkSystems NX-10) was 10 µm × 10 µm, and the scan rate was 0.3 Hz. The data were presented in 256 pixels. The shapes of the 3D-printed and solvent-cast films were observed using an optical microscope (CX23, Olympus Corporation, Shinjuku-ku, TYO, Japan) at 40× magnification. The digital image was corrected using Image View software.

### 3.6. Mechanical Properties of the ODFs

The mechanical properties of the ODFs were measured using texture analyzer TA XT2i (Stable Microsystems, Godalming, UK) equipped with a planar flat cylindrical probe (diameter: 4.8 mm, area: 18.1 mm^2^). This cylindrical probe could be widely used for determining the mechanical properties in an accurate and fast comparison of polymeric films [56]. Each film was placed in a sample holder with a cylindrical hole. Screws were fixed to a diameter of 6 mm. The measurements were performed by moving the probe vertically downward at a speed of 5.0 mm/s until the film was punctured at 25 ± 2 °C. Three replicates were performed for each kind of film.

### 3.7. HPLC Analysis

A 5 µm C18 (150 × 4.6 mm) column (Fortis^®^ Technologies Ltd., Neston, Cheshire, UK) was used, with the isocratic condition of mobile phase composition 40:60 (*v*/*v*) acetonitrile: triethanolamine buffer (5 mM, pH 3.5 with phosphoric acid). The standard solution was prepared by dissolving the ARP 10 mg in a volumetric flask with mobile phase as a diluent. The sample solution was quantitatively analyzed by comparing the peak area of the ARP standard solution. Subsequently, the mobile phase was degassed and filtered using a vacuum filtration system using a PTFE 0.45 µm membrane filter. The detection ultraviolet wavelength was set to 255 nm, the column temperature was 37 °C, the flow rate of the mobile phase was 1.0 mL/min, and the injection volume of the sample was 20 µL. The run time of each sample was 7 min.

### 3.8. In Vitro Disintegration Studies of the ODFs

The disintegration time of the film was measured using the Petri dish method [51]. After filling a 6 cm diameter Petri dish with 2 mL distilled water (37 ± 0.5 °C), the ODFs (2.2 × 2.2 cm) were placed on the water surface and shaken at 60 rpm to determine the disintegration time.

### 3.9. In Vitro Release Studies of the ODFs

The dissolution tests were performed at pH values of 1.2, 4.0, and 6.8 USP buffer using a PTWS-121C (Pharma Test, Siemensstraße, Hainburg, Germany) and the USP paddle II method [32,33]. The dissolution test temperature was 37 ± 0.5 °C, and the paddle speed was 75 rpm. The sampling times were 10, 20, 30, 45, 60, and 90 min, using a 1 mL sample volume. Before analysis, each dissolution sample was filtered by a 0.45 µm syringe filter (Merck Korea, Seoul, South Korea). The filtered sample solutions were stored in high-performance liquid chromatography (HPLC) vials.

## 4. Conclusions

We investigated an optimized polymer selection and manufacturing process for preparing ARP-loaded ODFs. The acceptable hardness and flexibility of the drug-loaded PVA and HPC filaments satisfied the processing requirements of the FDM technique in a 3D printer. ARP was amorphous in all drug-loaded filament and film formulations. The physicochemical and mechanical properties of the 3D-printed formulations supported the durability of film formation and the dissolution rate of ARP. AFM analysis demonstrated that the surface roughness of each different film was varied in the manufacturing process. The surface roughness of the films significantly affected the drug release rate. The in vitro dissolution performance of HPC is superior to that of PVA. The initial dissolution rate of the ARP-loaded 3D-printed film was higher than that of the solvent-cast film. The disintegration time of the ARP-loaded HPC 3D-printed film is shorter than that of the PVA 3D-printed film. Taken together, these studies suggested that 3D printing technology fused with HME technology can fine-tune the punctual strength of films, accelerating the water penetration and disintegration compared to the traditional solvent casting method while adhering to ideal standards of mechanical strength. The selection of the type of polymer and manufacturing process to additive manufacturing/3D printing could generate fast-disintegrating formulations to satisfy the unmet needs of patients with mental illness.

Drug-loaded filaments, successfully manufactured using the HME technology, could be used in 3D printing to prepare various solid dosage forms. The HME 3D printing process can be applied not only for the fabrication of ODFs but also for other biomedical applications, such as biomimetic 3D-printed scaffolds and personalized medical devices [57]. This study confirmed that the architectures of film formation had a significant effect on the specification of drug release according to the manufacturing process. Mechanical uniformity of pharmaceutical drug products can also be separately designed by each zone in a single unit. In fact, the filling density of solid dosage form is innovatively controlled by gradient modification to improve the release performance of drugs [58]. Regulatory guidance will focus on rigorously and efficiently examining the performance of complex architectures.

## Figures and Tables

**Table 1:** Composition and processing parameters of filaments prepared using the hot-melt extrusion technology.

Ingredient	HPC-Filament (wt%/wt%)	PVA-Filament (wt%/wt%)
Aripiprazole (ARP)	10	10
HPC	85	0
PVA	0	85
Citric acid	4	4
Sucralose	1	1
**Processing Parameter**	**HPC-Filament**	**PVA-Filament**
Screw speed (RPM)	100	100
Temperature (℃)	115	200
Feed rate (kg/h)	0.5	0.5
Screw configuration	Standard from ThermoFisher	Standard from ThermoFisher

**Table 2:** Investigation of mechanical properties of marked products, materials, and developed orodispersible films.

	Thickness (mm)	Drug Content (%)	Disintegration Time (s)	Puncture Strength (N/mm²)
HPC-3D Film	0.11 ± 0.02	98.07 ± 4.06	45 ± 3.5	0.65 ± 0.27
PVA-3D Film	0.11 ± 0.01	98.91 ± 3.10	63 ±10.2	0.93 ± 0.15
HPC-SC Film	0.12 ± 0.08	99.12 ± 2.06	62 ± 5.4	0.83 ± 0.30
PVA-SC Film	0.11 ± 0.07	98.41 ± 4.12	71 ± 12.3	1.12 ± 0.42
**Marketed Products** [45]				
EclipseFlash	0.048 ± 0.01			0.34 ± 0.02
SmartStrip	0.15 ± 0.02			0.40 ± 0.09
**References Materials** [45]				
Soft Tissue				0.10
Foil				0.97
Paper				1.32

**Table 3:** Morphological characteristics with the surface roughness of orodispersable films (3D, 3D printing; SC, solvent casting).

Surface Roughness Parameter	HPC-3D	PVA-3D	HPC-SC	PVA-SC
Roughness Average (nm)	85.007	27.748	33.274	8.748
Roughness Root Mean Square (nm)	102.868	35.025	44.310	12.117

## Data Availability

Data are contained within the article.
